# The Phylogeny of the Four Pan-American MtDNA Haplogroups: Implications for Evolutionary and Disease Studies

**DOI:** 10.1371/journal.pone.0001764

**Published:** 2008-03-12

**Authors:** Alessandro Achilli, Ugo A. Perego, Claudio M. Bravi, Michael D. Coble, Qing-Peng Kong, Scott R. Woodward, Antonio Salas, Antonio Torroni, Hans-Jürgen Bandelt

**Affiliations:** 1 Dipartimento di Genetica e Microbiologia, Università di Pavia, Pavia, Italy; 2 Dipartimento di Biologia Cellulare e Ambientale, Università degli Studi di Perugia, Perugia, Italy; 3 Sorenson Molecular Genealogy Foundation, Salt Lake City, Utah, United States of America; 4 Laboratorio de Genética Molecular Poblacional, Instituto Multidisciplinario de Biología Celular (IMBICE), La Plata, Argentina; 5 Armed Forces DNA Identification Laboratory, Armed Forces Institute of Pathology, Rockville, Maryland, United States of America; 6 Laboratory of Cellular and Molecular Evolution, and Molecular Biology of Domestic Animals, Kunming Institute of Zoology, Chinese Academy of Sciences, Kunming, China; 7 Laboratory for Conservation and Utilization of Bio-resource, Yunnan University, Kunming, China; 8 Unidade de Xenética, Instituto de Medicina Legal, Facultad de Medicina, Universidad de Santiago de Compostela, Grupo de Medicina Xenómica, Hospital Clínico Universitario, Santiago de Compostela, Galicia, Spain; 9 Department of Mathematics, University of Hamburg, Hamburg, Germany; University of Glasgow, United Kingdom

## Abstract

Only a limited number of complete mitochondrial genome sequences belonging to Native American haplogroups were available until recently, which left America as the continent with the least amount of information about sequence variation of entire mitochondrial DNAs. In this study, a comprehensive overview of all available complete mitochondrial DNA (mtDNA) genomes of the four pan-American haplogroups A2, B2, C1, and D1 is provided by revising the information scattered throughout GenBank and the literature, and adding 14 novel mtDNA sequences. The phylogenies of haplogroups A2, B2, C1, and D1 reveal a large number of sub-haplogroups but suggest that the ancestral Beringian population(s) contributed only six (successful) founder haplotypes to these haplogroups. The derived clades are overall starlike with coalescence times ranging from 18,000 to 21,000 years (with one exception) using the conventional calibration. The average of about 19,000 years somewhat contrasts with the corresponding lower age of about 13,500 years that was recently proposed by employing a different calibration and estimation approach. Our estimate indicates a human entry and spread of the pan-American haplogroups into the Americas right after the peak of the Last Glacial Maximum and comfortably agrees with the undisputed ages of the earliest Paleoindians in South America. In addition, the phylogenetic approach also indicates that the pathogenic status proposed for various mtDNA mutations, which actually define branches of Native American haplogroups, was based on insufficient grounds.

## Introduction

America was the last continent to be colonized by humans, and molecular data provided by different genetic systems [1], [2] have been extensively employed to shed light on the routes and times of human arrival and dispersion into the New World. As for mitochondrial DNA (mtDNA), it has been clear, since the early nineties, that mtDNAs of Native Americans could be traced back to four major haplogroups of Asian origin shared by North, Central and South American populations [3]–[7]. These were initially named A, B, C and D, and are now termed A2, B2, C1 and D1 [8]. Afterwards, a fifth haplogroup – now known as X2a – was described in Native Americans, but in contrast to the four “pan-American” haplogroups, its geographic distribution is restricted to some Amerindian populations of northern North America [8]–[12]. Later, two more haplogroups – D2a and D3 – were identified: D2a in the Aleuts and Eskimos [13], [14] and D3 only in the Eskimos [15], [16]. Most recently there were two further (uncommon) additions – D4h3 and C4c [14], [17] – bringing the total number of Native American haplogroups to nine.

Since the early studies, the interpretation of mtDNA data has been rather controversial with scenarios postulating one to multiple migrational events from Beringia at very different times (between 11,000 and 40,000 years ago) (for a review, see [7]). Pinpointing an accurate timeframe for the arrival of the Native American founders would be essential to solve such a debate. Yet, accurate ages can only be based on large numbers of complete mitochondrial genomes, and American mtDNA haplogroups were only poorly represented in the total database of >3000 complete mtDNA sequences until very recently. Thus, despite the protagonist role of Native American mtDNAs in high-resolution mtDNA studies 15 years ago [4], America remained the continent from which we had the least information about the sequence variation of entire mtDNAs. Worse, the available information had to be retrieved from the web in a hit or miss fashion and suffers in part from improper documentation, oversights, and inadvertent nomenclature (Text S1). The overall situation is now beginning to change with some new data available in literature and public databases [14], [18], [19], but the interpretation of subsets of these data continue to remain controversial. For instance, the work by Tamm et al. [14] suggests that the Asian ancestors of the first Native Americans paused when they reached Beringia and that their (swift) migration southward might have occurred only ∼13,500 years ago.

Among the novel mtDNA sequences, there are 265 from “Hispanics” and “African-Americans” that recently became available in GenBank [19]. A survey of their variation reveals that 101 mtDNAs of Native American origin were included (47 belonging to haplogroups A2, 13 to B2, 30 to C1, and 11 to D1). Those mtDNAs are not associated with either a specific Native American population/tribe or a specific geographic region but are undoubtedly of Native American origin. Furthermore, due to the fact that these are all from individuals living in the US, they probably provide a fairly good overview of the mtDNA pool of extant or extinct Native American populations from North and Central America plus the Caribbean (due to the contribution of Mexicans, Puerto Ricans, Cubans, Salvadorans, etc. to the present-day US American population), and their analysis might provide important new clues about the process of human colonization of the Americas and the origin of Native Americans. Thus, the aim of this paper is not only to (i) perform a comprehensive analysis of all available complete (or almost complete) sequences of Native American ancestry belonging to the four major pan-American haplogroups, (ii) identify their internal clades and candidate founder sequences, and (iii) estimate their expansion times into the Americas, but also to (iv) provide a framework on which future phylogeographic studies, which remain scarce, can build upon.

## Results

### The phylogeny of pan-American haplogroups A2, B2, C1, and D1

**Figure 1:**
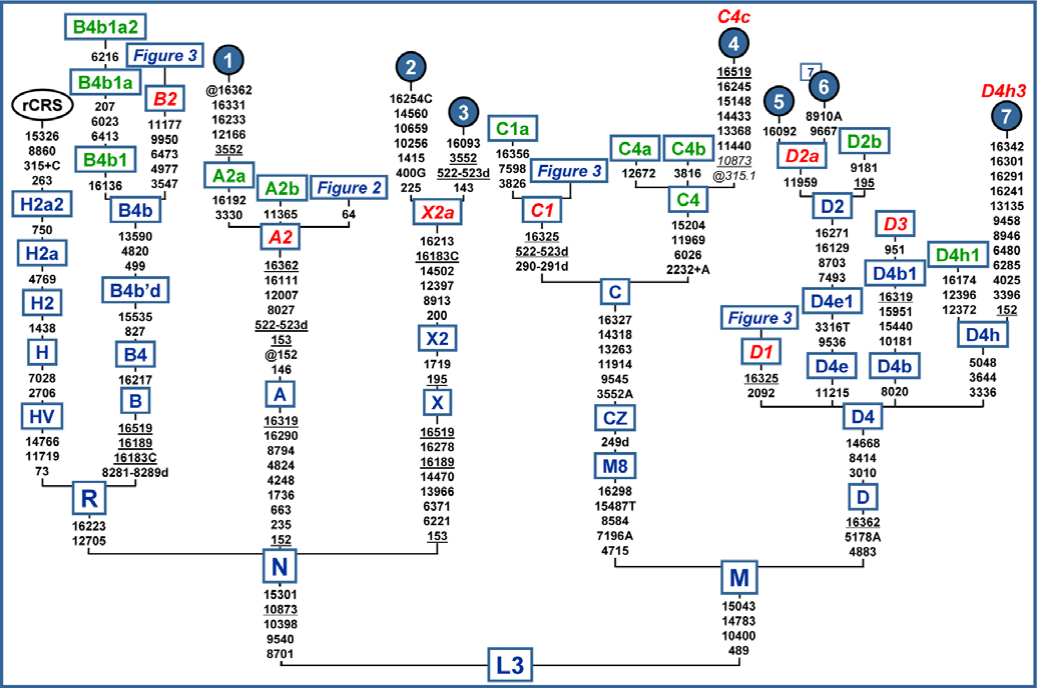
Basal tree encompassing the roots of Native American mtDNA haplogroups. The tree is rooted on the haplogroup L3 founder and the position of the revised Cambridge reference sequence (rCRS) [49] is indicated for reading off sequence motifs. Closely related Asian branches are indicated in green. Detailed phylogenies for the four pan-American haplogroups (A2, B2, C1, and D1, highlighted in red) are shown in the corresponding figures. The complete sequences that are currently available for the other four Native American haplogroups (X2a, C4c, D2a, and D4h3, highlighted in red) are also displayed. Haplogroup D3 is common among Inuit populations [16], but all complete sequences available are from Siberia [13], [18]. As for A2a, the HVS-I motif (16111 16192 16223 16233 16290 16319 16331) of the reported sequence (no. 1) is common in Na-Dené groups [5]. Sequence no. 2 has been revised taking into account that the originally reported transitions at 4732 and 5147 [8] were artifacts due to a sample mix-up, while sequence no. 6 represents the shared motif of six Aleutian mitochondrial genomes [13]. Mutations are transitions unless specified: suffixes indicate transversions (to A, G, C, or T) or indels (+, d). Mutations back to the rCRS nucleotide are prefixed with @. Recurrent mutational events are underlined. Mutations in italics are either disease-causing or heteroplasmic or likely erroneous (and do not enter age calculations). We have followed the recent guidelines for standardization of the alignment in long C stretches [50], but disregarded any length variation in the C stretches that would then be scored at 309 or 16193 (which is often subject to considerable heteroplasmy). A number flagging a circled haplotype indicates the number of individuals sharing the corresponding haplotype (if >1). Additional information is provided in Text S4, while Table S1 lists the source of the complete genomes.

To define the phylogeny of A2, B2, C1, and D1 at the highest level of molecular resolution – that of complete mtDNA sequences, it is necessary to evaluate (and possibly to expand) the current data set of published mtDNA sequences in regard to reliability as well as to update and correct the nomenclature (Text S1). Figure 1 displays the roots of A2, B2, C1 and D1, together with the complete sequences belonging to the much less common Native American haplogroups C4c, D2a, D3, D4h3 and X2a [8], [9], [12]–[15], [20]. Moreover, for a better discrimination from closely related Native American counterparts, some Asian (or Beringian) branches (B4b1a2, A2a, A2b, C1a, C4a, C4b, D2b, and D4h1) are illustrated. As for the phylogeny of haplogroup A2, we maintain the codes A2a and A2b for the circumpolar branches [16]. For branch A2a with the characteristic C16192T transition in HVS-I (which on its own is insufficient to identify a haplogroup because it is highly recurrent throughout the mtDNA phylogeny), coding-region information is now available revealing the additional diagnostic marker C3330T [14], [18].

**Figure 2 pone-0001764-g002:**
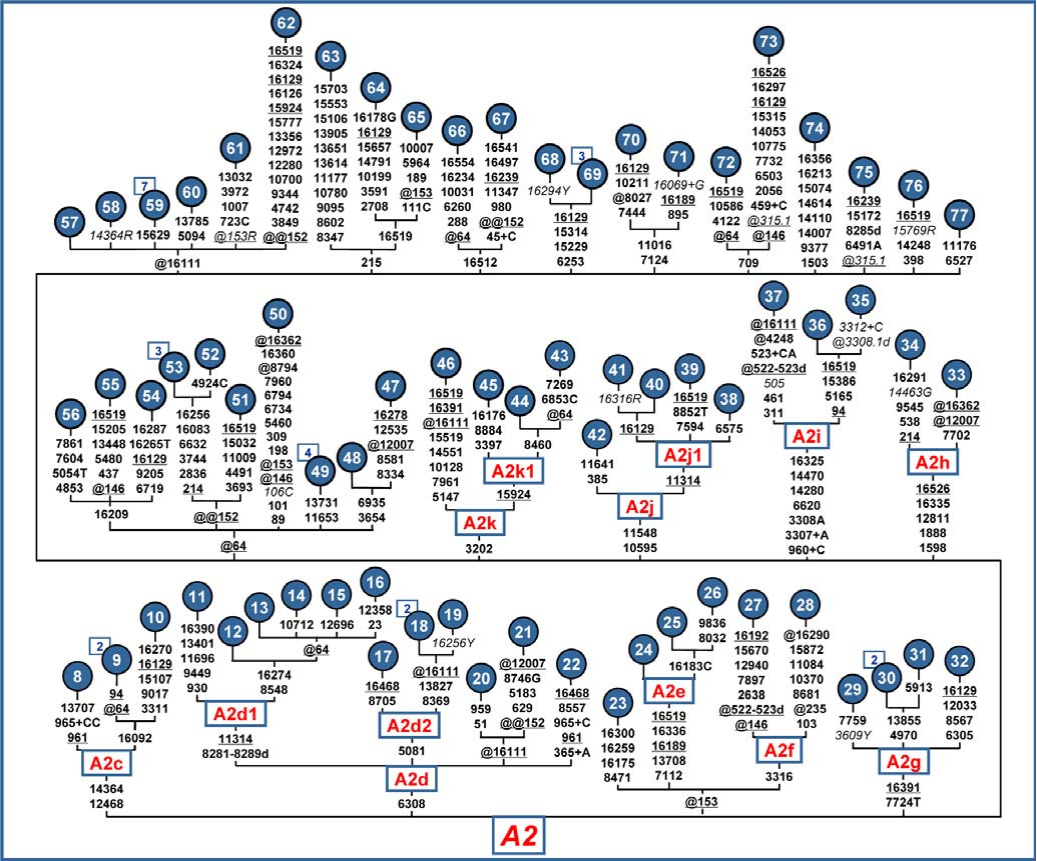
Phylogeny of complete mtDNA sequences belonging to haplogroup A2. The sequencing procedure for the novel complete sequences and the phylogeny construction were performed as described elsewhere [47]. Recurrent mutational events within the haplogroup are underlined, while mutations in italics are either disease-causing or heteroplasmic or likely erroneous, and were not used for age calculations. Table S1 lists the source of the complete genomes. For additional information, see the legend for Figure 1.

**Figure 3:**
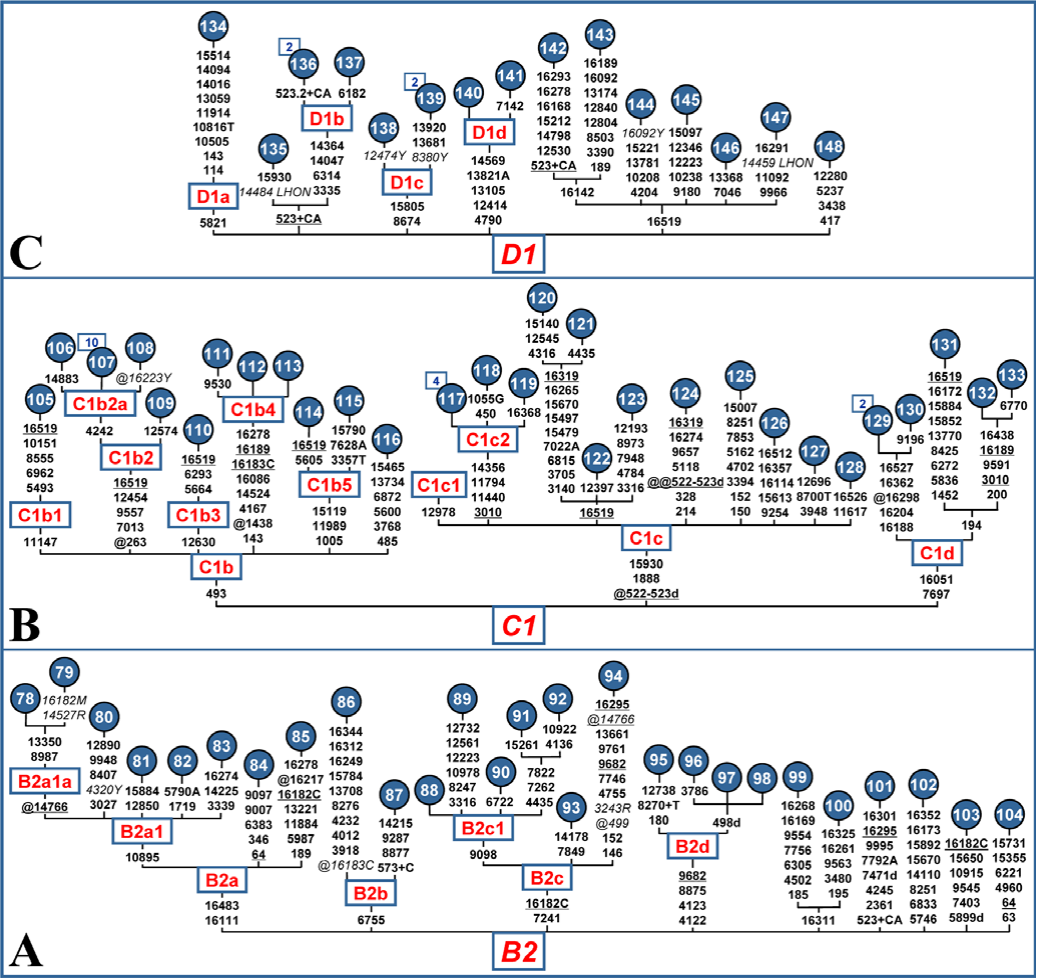
Phylogeny of complete mtDNA sequences belonging to haplogroups B2 (A), C1 (B) and D1 (C). For additional information, see the legends for Figures 1 and 2.

The complete variation of all available mtDNA sequences belonging to haplogroups A2, B2, C1, and D1 is displayed in the phylogenies of Figures 2 and 3. As for the phylogeny of A2 (Figure 2), we rename the “A2a” and “A2b” branches of Accetturo et al. [21] as A2d and A2e, maintaining the definition of A2c for the branch with the motif T12468C-G14364A. Moreover, we define six novel branches (A2f - A2k) based on all available information for haplogroup A2 (Table S1) and [20], [22]. Numerous independent back mutations at nucleotide positions (nps) 64, 146, 152, 153, 16111, and 16362 are evident (that on their own do not justify support for subhaplogroup naming). Many HVS-I and HVS-II lineages from haplogroup A2 reflect this seemingly mosaic feature of instability. Some additional information on the population distribution of the subhaplogroups can also be drawn from the early high-resolution RFLP data [5], [23] and an extensive database of published control-region sequences (mainly comprising HVS-I) (Text S2).

The phylogeny of haplogroup B2 (Figure 3A) reveals at least four subhaplogroups (B2a - B2d). B2a is defined by the control-region transitions C16111T and G16483A, while the sub-branch B2a1 is defined by the coding-region transition A10895G previously seen as a *Taq*I site at 10893 in haplogroup B mtDNAs from the Navajo, Ojibwa, and Pima [5]. The branches B2b and B2c are based on the presence of transitions G6755A and A7241G, respectively. B2c was also identified as a *Rsa*I site at 7241 in two mtDNA haplotypes from Mexico [23], while its sub-branch B2c1 seems to be defined by a transition at np 9098. The branch B2d (coding-region motif 4122-4123-8875-9682) is probably rather widespread in lower Central America since it was found in the Wayuùs and Ngöbes [14] and (as a *Hae*III gain at np 8872) in several other Chibchan-speaking populations [23], [24].

As for haplogroup C1, all sequences appear to fall into one of the three subhaplogroups C1b, C1c, and C1d (Figure 3B). These are most likely spread all over the Americas. Indeed, the transitions at nps 493 and 16051 that define C1b and C1d, respectively, have been observed in haplogroup C1 control-region motifs from a wide range of Native American populations, including some from the southern part of South America. For C1c, which lacks basal salient HVS-I or RFLP motifs, its presence in South America is confirmed by its detection in Colombia [14] and the observation that South American C1 mtDNAs are not fully covered by subhaplogroups C1b and C1d [25], and thus the remaining C1 lineages likely belong to C1c. These findings support the scenario that C1b, C1c and C1d (and their distinguishing mutational motifs) most likely arose early – either in Beringia or at a very initial stage of the Paleoindian southward migration [14].

As for D1 (Figure 3C), the basal mutation of D1a (sequence #134) is based on the comparison with four coding-region sequences (Am02, 10, 11, 14) reported by Kivisild et al. [26]. The three additional sub-clades, D1b, D1c, and D1d have been defined by using either the novel sequences reported in this study or those from Parsons [19].

Overall, the four phylogenies appear to be quite starlike, especially the B2 and D1 trees having high indices (∼0.5) of starlikeness (Table 1). In the case of haplogroup C1, the three basal branches (C1b, C1c, and C1d) are themselves starlike, with the exception of C1b where a very low index of starlikeness (influencing also C1) is mainly due to an over-sampling (10 instances) of the root haplotype of the sub-branch C1b2a (sequences #107). The significance of starlike patterns in the Native American haplogroups would be that the successful propagation event of these haplogroups and some of their major branches (in Beringia or later on the move further south) can very well be dated assuming a reliable calibration of the mtDNA mutation rate. The point estimates for the coalescence times of haplogroups A2 (without the branches A2a and A2b), B2, C1 (without the Asian branch C1a), and D1 yield 18.1±1.8, 21.2±2.4, 23.8±4.3, and 18.6±2.3 ky, respectively, based on all 219 coding-region sequences (Table 1) and by employing the calibration of 1 coding-region substitution every 5,140 years [27]. The haplogroup ages thus fall into the range of 18–24 ky with an average of about 20.2 ky (Table 1). This value is a little bit lower (∼19.0 ky) if the roots of the three branches of C1 (C1b, C1c and C1d), instead of C1 as a whole, are considered as Native American founders. This might be a (slight) underestimation because C1d is clearly under-represented in this study (comprising only eight mtDNAs). Thus, excluding C1d, the time frame is restricted to 18–21 ky and these estimates are about 1.4-fold higher than the larger time frame of 11–17 ky (A2: 13.9±2.0 ky; B2: 16.5±2.7 ky; C1b: 14.7±4.7 ky; C1c: 15.8±4.7 ky; D1: 10.8±2.0 ky) that was recently estimated [14] in a smaller dataset (105 mtDNA sequences) adopting a different calibration [26].

**Table 1:** Haplogroup coalescence time estimates

Haplogroup	No. (*n*) of mtDNAs^a^	No. of base sub-stitutions^a^	*ρ* b	*σ* c	Star-likeness^d^ *ρ/*(*nσ* ^2^)	T (years)^e^	ΔΤ (years)
**A2**	96^f^+1	321+3	3.340	0.322	0.332	17,200	1,700
**A2 (w/o A2a, A2b)**	86+1	304+3	3.529	0.348	0.335	18,100	1,800
**B2**	27+16	116+61	4.116	0.463	0.447	21,200	2,400
**C1 (w/o C1a)**	42+13	198+57	4.636	0.836	0.121	23,800	4,300
**C1b**	21+4	86+14	4.000	1.150	0.121	20,600	5,900
**C1c**	15+7	63+23	3.909	0.695	0.368	20,100	3,600
**C1d**	6+2	13+4	2.125	0.573	0.809	10,900	2,900
**D1**	17+17	67+56	3.618	0.441	0.547	18,600	2,300
**Total ** g ** (A2,B2,C1,D1)**	172+47	684+177	3.932	0.311	0.186	20,200	1,600
**Total ** g ** (A2,B2,C1b,C1c,C1d,D1)**	172+47	649+161	3.699	0.274	0.225	19,000	1,400

a First summand refers to the complete mtDNA sequences displayed in Figures 2 and 3 and second summand refers to additional entire coding-region sequences [1]–[3]. Three C to G transversions (at positions 14974, 15439, and 15499) [1] – likely candidates for phantom mutations [2] that went undetected – were disregarded.

b The average number of base substitutions in the mtDNA coding region (between positions 577 and 16023) from the root sequence type.

c Standard error calculated from an estimate of the genealogy [4].

d Starlikeness (“effective star size” [4]) can take values between 1/*n* (single haplotype representing *n* mtDNAs) and 1 (perfect star phylogeny).

e Estimate of the time to the most recent common ancestor of each cluster, using an evolutionary rate estimate of 1.26±0.08×10^−8^ base substitutions per nucleotide per year in the coding region [5], corresponding to 5,140 years per substitution in the whole coding region.

f This includes one Apache A2a mtDNA (#1 in Table S1) and 9 Siberian mtDNAs (four A2a and five A2b) [6], [7].

g Without A2a and A2b mtDNAs.

### Detrimental mtDNA branches in Native Americans?

In some of the newly defined Native American branches, one can identify mutations for which a pathogenic role was suggested in the medical literature. The seemingly ‘detrimental’ status of mutations G3316A and G13708A, defining haplogroups A2f and A2e respectively, has already been questioned and discussed at length in the East Asian mtDNA context [28]. The occurrence of both mutations is not infrequent (also appearing, for instance, in haplogroups B2 and D1) and therefore, not unexpectedly, they participate in the motifs of several haplogroups. A similar case is represented by the transition T1005C, which was proposed as a primary mutation for non-syndromic hearing loss [29], [30], and defines for instance the Asian haplogroup F2. In the context of Native American haplogroups, T1005C appears as a basal mutation of C1b5 – a branch of haplogroup C1b. Thus, all of these mutations are old and have been transmitted for at least some hundreds of generations. Although an effect of “old” mtDNA mutations in some multi-factorial/complex (and common) diseases cannot be ruled out *a priori*, a pathogenic role specific for such variants can, however, only be inferred from association studies in which haplogroup frequencies are properly evaluated in both patients and controls [31].

An extremely interesting case of a mutational motif marking a Native American branch of the mtDNA phylogeny is represented by the T3308A transversion with a subsequent insertion of one C (3308+C) that characterize haplogroup A2i. The insertion, first reported in a patient with dystonia, leads to a frameshift mutation for which a pathogenic role was proposed [32]. However, the other mutation of the motif – the T3308A transversion – eliminates the starting codon (methionine) of the ND1 subunit by converting it to lysine, thus paralleling the scenario first described for the T3308C transition that marks the African haplogroup L1b [33]. The finding that the elimination of the methionine codon AUA at position 1 of the ND1 subunit is polymorphic in some populations clearly indicates that the maintenance of that codon is not essential in our species, and therefore the insertion of one C at 3308 does not cause a frameshift for the entire gene. This is most likely due to the fact that the third codon (AUG) of the ND1 subunit also encodes for methionine, thus despite the shortening of two amino acids, ND1 could still retain its function.

A different case is the one concerning the homoplasmic mutation T9205C detected in one mtDNA (no. 54) belonging to haplogroup A2 (Figure 2). This mutation converts the termination codon of the ATPase 6 subunit into a glutamine codon, and extends the subunit by ten amino acids (Gln-Trp-Pro-Thr-Asn-His-Met-Pro-Ile-Met) at the carboxyl-terminus. No information is available concerning the health/disease status of the subject harboring this mitochondrial genome. Thus, for the moment it cannot be ruled out that T9205C is a benign or mildly deleterious variant, despite the considerable extension of the amino acid chain. Such a scenario would parallel the situation previously reported for mutations A7443G, G7444A, and A7745C, which erase the termination codon of the COI subunit and whose pathogenic role is unclear [34].

Another illustrative case of hypothesized association between mtDNA mutations and a complex disorder is represented by the G1888A transition which could play some role in the pathogenesis of Type 2 diabetes [35] – a scenario that would be compatible with the well-known common-disease/common-polymorphism hypothesis. This transition is characteristic of both A2h and C1c, but is also present in West Eurasia, mainly in haplogroup T [36], and in South Asia, mainly on haplogroup M5 [37]. Unfortunately, the study of [35], similar to the most recent work [38], which again implicitly targeted haplogroup T, is absolutely insufficient to shield against population substructure influencing patient cohorts and control subjects in different ways. Especially in a country such as Brazil, matrilineal population substructure matters a lot across the country [39], as well as across social strata, which often correlate with continental matrilineal ancestry. Case-control association studies that do not consider the haplogroup context in which observed mutations are embedded do not allow an objective evaluation of the role played by mtDNA variants in disease expression either, because additional variables (such as social strata and ethnicity) may influence haplogroup frequencies (Text S3) [40].

## Discussion

The estimated ages (18–24 ky) of the four pan-American haplogroups A2, B2, C1, and D1 are quite similar with an average value of 20 ky. Thus, if A2, B2, C1, and D1 entered the Americas without variation in the coding region – in other words, each with only a single (successful) founder sequence (the root haplotype), their entry into the Americas would have occurred right after the peak of the Last Glacial Maximum (LGM, centered at ∼21.0 kya and extending from 19.0 to at least 23.0 kya [41]), or slightly earlier, so that a coastal (Pacific) route would have been the only option during such glacial periods. On the other hand, it is quite plausible that some intra-haplogroup variation – hardly noticeable at the level of HVS-I motifs – already existed in Beringia and was carried directly further south into the American double-continent. If one assumes that at least the root haplotypes of A2, B2, D1, as well as of C1b, C1c, and C1d were of Beringian origin, then the entry time would come slightly down (19.0 kya), that is, falling exactly at the end of the LGM. Moreover, the relatively lower coalescence time (∼17 ky) of the entire haplogroup A2 (Table 1) – including the shared sub-arctic branches A2b (Siberians and Inuits) and A2a (Siberians, Inuits and Na-Dené) [5], [14], [16], [18] – is probably due to secondary expansions of haplogroup A2 from Beringia long after the end of the LGM, which would have averaged the overall internal variation of haplogroup A2 in North America – the main source of the A2 mtDNAs in this study.

In any case, all the abovementioned scenarios do not support the ‘Clovis-first’ hypothesis, but are well in agreement with the undisputed ages of the earliest Paleoindians in South America [42]. This conclusion would not change if one adopted the effectively faster rate of Kivisild et al. [26] based only on synonymous substitutions, which would generally shrink ages by a factor of ∼3/4, as judged from a comparison with both the ages of the Native American haplogroups [14] and those of super-haplogroups L, L3, M, and N [43]. Therefore the main difference between both rates seems to concern only the absolute calibration as manifested in the estimated global coalescence times for super-haplogroup L. It is dubious whether the partial utilization of the coding-region information [14], [26] leads to more credible age estimates, taking into account the extremely low amount of synonymous mutation data characterizing younger clades, such as the Amerindian ones, and the extreme discrepancies with ages based on control-region variation of some haplogroups such as H, I, T, and U5 [44]. Moreover, if as suggested [26], the molecular clock did not apply to the entire coding region, but only to the synonymous mutations in the 13 genes coding for protein subunits, it would be rather unlikely that an age overlapping such as that reported for the well represented founder haplogroups (A2, B2, D1, C1b, and C1c) in Table 1 would be observed. In any case, with both clocks, a Beringian stage preceeding the expansion into the Americas – estimated at slightly different starting times and with a different duration depending on the clock employed – most likely took place, thus explaining the differentiation of the pan-American lineages from the Asian sister-clades (Figure 1).

Our snapshot of the phylogenies for haplogroups A2, B2, C1, and D1 is only partially representative of Native American mtDNA variation, since most likely it only marginally includes the variation of Native American populations from Central and South America. However, despite this limitation, it is clear that one has to anticipate a pronounced starlike pattern near the root of each respective founder haplogroup/branch. The starlike pattern enhances the precision of the dating of the human entry into the Americas, but inevitably hinges upon the calibration employed and, perhaps more importantly, on a detailed founder analysis across the double-continent. Therefore it will require major sampling and sequencing efforts in the future for uncovering all of the most basal variation in the Native American mtDNA haplogroups by targeting, if possible, both the general mixed population of national states and autochthonous Native American groups, especially in Central and South America.

A widespread knowledge of the specifics for the Native American haplogroups can also prevent the publishing of effectively mutilated or distorted mtDNA sequences from complete sequencing efforts in clinical studies [45], [46], but most importantly, the dissection of pan-American haplogroups into clades of younger age and more limited geographic and ethnic distributions is essential for reliable association studies between mtDNA haplogroups and complex disorders [31].

## Materials and Methods

The source of the sequence data (171 complete mtDNA sequences) employed for the phylogeny construction are listed in Table S1 (and Text S5), together with 14 novel Native American mtDNA sequences (four each belonging to haplogroups A2 and C1; three each belonging to B2 and D1) from the Dominican Republic (N = 4), Canada (N = 3) and United States (N = 7). The latter were completely sequenced as described elsewhere [47]. Additional 47 entire coding-region sequences [20], [26] were employed only for time estimation and inference of branching nodes (see also Text S4).

The 101 complete mtDNA sequences [19] represent 13 of the 18 most common HVS-I & II haplotypes among the “Hispanic” component of the SWGDAM database [48]. Anonymous, unrelated samples were identified and obtained from either an internal Armed Forces DNA Identification Laboratory (AFDIL) database, or from 575 regional “Hispanics” living in the southern and northeastern regions of the US. The control region of their mtDNAs was then sequenced in order to determine the common HVS-I & II haplotypes [19].

### Electronic database information

Accession numbers and URLs for data presented herein are as follows: GenBank, http://www.ncbi.nlm.nih.gov/Genbank/ (for the 14 novel complete mtDNA sequences [accession numbers EF079873-EF079876; EU431080-EU431089]); (for sequence no. 3 of Figure 1 [accession number EU439939])

## Supporting Information

Text S1 Mistakes, phantom mutations and discrepancies in literature and public databases
(0.06 MB DOC)

Text S2 Further information from mtDNA control-region and RFLP data
(0.08 MB DOC)

Text S3 Additional information concerning mtDNA disease studies
(0.04 MB DOC)

Text S4 Additional information for Figures 1–3
(0.04 MB DOC)

Text S5 Additional references
(0.04 MB DOC)

Table S1 Source of the complete mtDNA sequences
(0.39 MB DOC)
