# Supplementary material for: The Phylogeny of the Four Pan-American MtDNA Haplogroups: Implications for Evolutionary and Disease Studies
Source: PLoS One. 2008 Mar 12;3(3):e1764. doi: 10.1371/journal.pone.0001764 (PMC2258150; doi:10.1371/journal.pone.0001764)
Supplement: Text S1 — Mistakes, phantom mutations and discrepancies in literature and public databases (0.06 MB DOC) [file pone.0001764.s001.doc]

# Text S1. Mistakes, phantom mutations and discrepancies in literature and public databases

*Phylogenetic studies.*

The geneticist or anthropologist who wishes to study the mtDNA variation in individuals of matrilineal Native American ancestries would have at the moment a very difficult job. He/she might come across the paper [1] that provides a basal classification scheme and supplementary information on previously published coding-region sequences [2,3], but the corresponding complete mtDNA sequences are difficult to retrieve (see notes to Table S1). The most recent submission (July 14, 2007) of the data from [2] to GenBank (Acc. No. EF657231 to EF657790) may additionally confuse the user as all the phantom mutations that were announced in [3] to be removed are still in place, e.g. the transversions to G at positions 7927, 7985, 11227, 14227, 14385, 14460, and 15040 in the Native American coding-region sequences no. 171, 174, 200, 329, and 377 [2]. In the lack of any properly published revision, it is not even clear whether the transversions at 14463 in no. 171 (haplogroup A2; see #34 in our Table 1 below) and at 14974 in no. 197 (haplogroup B2) present in the "revised" data [3] that we employed would be real or not. Moreover, in the study [1] and in the mtDB database [4] the continental assignments of the mtDNAs are not always correct. In particular, the distinction between Asian and Native American mtDNAs was blurred. As for the most recent data of Tamm et al. [5], there are some minor discrepancies between the sequences read off from their Fig. S1 and from the corresponding sequences stored in GenBank (which we have consistently used).

To exacerbate the situation, a genuine Native American branch (subhaplogroup) of haplogroup C1 slipped into the Asian mtDNA phylogeny under the name “C6” in the recent update of the East Asian mtDNA phylogeny [6] due to the lack of control-region information from the sequence data employed. Finally, although prior to November 2006 no additional complete sequences were published, the naming of new branches has been going on – but based only on the poor information that hypervariable segment I (HVS-I) sequences can provide and, unfortunately, by baptizing nested branches of A2 as A3, A6, A7, and A8 [7] instead of following the standard nomenclature rules for naming nested branches, starting with A2a, etc. [8]. It still remains very unlikely that haplogroup B4b1a2, which was called B1 by [9] at the time, could be a Native American branch. The T16136C transition on a haplogroup B4 is, for instance, rather common in the Japanese, Korean, Chinese, and Guam fractions of the SWGDAM mtDNA database [10], but is completely absent in the US American fractions (“Caucasian”, “African-American”, and “Hispanic”). Furthermore, at the level of the entire American double-continent, only a single occurrence (sample #AND19, a Peruvian Quechua from Arequipa) is recorded in the literature bearing the transition T16136C diagnostic for the B4b1 clade [11].

*Disease studies.*

The study of [12] provided two haplogroup A2 sequences (control #7 corresponding to sequence #50 in our Figure 2, and patient #12) without listing the nucleotide variants that exist between the rCRS and the root of super-haplogroup N; moreover, the C8794T change is missing in one case (control #7). Similarly, the study [13] determined only a 2/3 subset of the mutation motif separating rCRS from the root of haplogroup A (but reported well all additional mutations for A2 status).
