# Supplementary material for: The Phylogeny of the Four Pan-American MtDNA Haplogroups: Implications for Evolutionary and Disease Studies
Source: PLoS One. 2008 Mar 12;3(3):e1764. doi: 10.1371/journal.pone.0001764 (PMC2258150; doi:10.1371/journal.pone.0001764)
Supplement: Text S2 — Further information from mtDNA control-region and RFLP data (0.08 MB DOC) [file pone.0001764.s002.doc]

**Text S2. Further information from mtDNA control-region and RFLP data**

*Haplogroup A2.*

The characteristic A2b mutation T11365C is recognizable by a loss of the 11362 *Alu*I site [14]. Despite the fact that it was first described as distinctive of the subhaplogroup named “A2a” [15], we maintained the now commonly used nomenclature for A2a and A2b [16]. The G15172A transition (see #75 in Figure 2) causes the *Hae*III site loss at 15172, a site which was indeed observed in RFLP types AM11 and AM106 from Mexico. Furthermore, the A11548G transition in A2j leads to –11546 *Rsa*I, which was recorded in types AM100, AM103, and AM104 (also sampled in Mexico). One further match between the complete A2 sequences and the high-resolution RFLP haplotypes is found for sequence #27 (A2f) in Figure 2 and haplotype AM62 described in ~36% of the Ojibwa from North America [17]; in fact, transitions at positions 2638, 3316 and 7897 correspond to *Hae*III changes at 2636 (site gain) and 3315 (site loss), and a *Rsa*I site loss at 7897 [17], respectively. Moreover, the two mutations defining sub-branch A2d1 are supported by the Mexican type AM111: the corresponding *Alu*I site gain at 11313, however, was incorrectly assigned 8 bp apart.

Sequence #50 in Figure 2 of [12] is a member of a conspicuous set of lineages present in populations from Lower Central America whose ancestral control region motif can be defined by the presence of C16360T and a 6-bp deletion involving positions 106-111 on top of the A2 root. This deletion abolishes a recognition site for *Msp*I at position 104, which has been reported in the Boruca, Kuna, Guaymi, Bribri-Cabecar [17], and the Teribe [18]. Although moderately recurrent, it has always been seen linked with a C16360T transition in Chibchan-speaking populations from Central America [17,19-21]. Sequence #50 carries a derived motif in its HVS-II segment, namely the co-occurrence of transitions T89C and C198T on top of the A2 plus C16360T motif, a combination that hitherto has only been observed in the Huetars and Ngöbe from Central America [21,22]. However, sequence #50 lacks the otherwise expected 6-bp deletion and offers instead the 106C transversion. Since the reversal of a 6-bp deletion is highly unlikely, an incorrect interpretation might have occurred.

*Haplogroup C1.*

The transition at 7013 (sub-branch C1b2) was found as 7013 *Rsa*I site loss in several Amazonians [17], while the sub-branch C1b3 has the characteristic 12630 transition, which is also recognized by a restriction site, namely by –12629 *Ava*II, as seen in AM120 from Mexico [23]. Also the variant G11440A of C1c2 has been previously seen in Native Americans from Mexico as +11439 *Mbo*I site [23].

The only C lineage from [2], other than the haplogroup C5 members [1], for which C1 status cannot be inferred by comparison with shared mutations with complete C1 sequences is sample no. 174, having the private transition motif A5894G-G6261A-A10397G-G13813A-T14215C. However, the RFLP data [17,23] provide a hint. The A10397G transition (which appears to be a rather infrequent mutation) would erase both the 10394 *Dde*I and 10397 *Alu*I restriction sites. In fact, three RFLP types found in Mexico (AM30, AM31, and AM122) testify to this reverted RFLP pattern. This justifies our assumption that one can also consider sample no. 174 as of Native American ancestry, which likely is a member of C1b since this branch is only recognized by a control-region mutation (A493G) in contrast to the other two Native American branches of haplogroup C1.

Since haplogroup C1 as a whole and its subhaplogroup C1b are only characterized by mutations in the control region, trees that are exclusively based on the coding region [2,24] are not helpful for uncovering the phylogenetic relationships within haplogroup C1. This also concerns the huge MITOMAP mtDNA tree [25], where two apparently polyphyletic clusters emerged within the C branch due to homoplasy at the highly variable positions 1719 and 15930 (Table 6 in [24]).
