# Supplementary material for: The Phylogeny of the Four Pan-American MtDNA Haplogroups: Implications for Evolutionary and Disease Studies
Source: PLoS One. 2008 Mar 12;3(3):e1764. doi: 10.1371/journal.pone.0001764 (PMC2258150; doi:10.1371/journal.pone.0001764)
Supplement: Text S3 — Additional information concerning mtDNA disease studies (0.04 MB DOC) [file pone.0001764.s003.doc]

**Text S3. Additional information concerning mtDNA disease studies**

In the context of the common-disease common-polymorphism hypothesis, any variant could be considered as a candidate contributing to some common disease, independent of its age and frequency in the population. However, indiscriminate case-control association studies of mtDNA variation can easily lead to premature claims of pathogenicity if just ‘random’ control samples are taken that were not controlled for population substructure. This is especially true in the context of mtDNA studies where one must consider the fact that mtDNA variation is strongly structured in populations. Thus, it is puzzling to learn that several basal mutations in A2 and C1 were assumed to have some detrimental effect as inferred from studies with improper designs. In the extreme case, an entire Native American haplogroup, such as haplogroup B2, could easily become the target of disease association. For example, the Ile to Val amino acid change in *ND1* due to A3547G – a marker of all haplogroup B2 lineages – was considered in a study that aimed at identifying mutations predisposing to Parkinson’s disease (PD), but finally “*no difference was noted in the frequencies of the 3547 mutation in PD and control subjects*” [26]*.* Even more extreme is the claim that the mutation A827G, characteristic of haplogroup B4bd in which B2 is nested (Figure 1), is pathogenic [27]; see a reassessment by [28].

Most of the mutations mentioned here that were suspected of disease association (A827G, T1005C, G1888A, T3308A, 3308+C, G3316A, A3547G, T12338C, and G13708A) had their genesis in single case studies because the published record was not consulted and/or MITOMAP [25] had not listed those mutations as polymorphisms at the time. Now (as of December 17, 2007) all those mutations except for T3308A and 3308+C are listed as “MtDNA Coding Region Sequence Polymorphisms” in MITOMAP. In addition, A827G and T1005C also appear there under the category “Reported Mitochondrial DNA Base Substitution Diseases: rRNA/tRNA Mutations” with status “Under Review”, G3316A there as “Unclear”, and G13708A with the comment “that some published reports have determined the mutation to be a non-pathogenic polymorphism”, thus, no green light from MITOMAP either for disease status.

In fact, several Native American haplogroups bear mutations that could increase the suspicion of disease status while also paralleling that of sub-Saharan African haplogroups, such as L1b as a whole in regard to MELAS [29] or a specific (unnamed) branch of L2a1 with elevated ‘risk’ of prostate cancer [30]. Without a detailed knowledge of all haplogroups – major or minor – thriving in the geographic region or ethnic population of interest, one cannot evaluate whether a mutation defining a particular minor subhaplogroup is responsible for the observed statistical association. Simply screening single coding-region sites believed to participate in the etiology of a disease without properly monitoring confounding variables such as population stratification could easily become a “lottery” since the frequency of a certain haplogroup in the area could actually constitute the major variable determining a strong correlation with the disease in a case-control association study.
