# Supplementary material for: The Phylogeny of the Four Pan-American MtDNA Haplogroups: Implications for Evolutionary and Disease Studies
Source: PLoS One. 2008 Mar 12;3(3):e1764. doi: 10.1371/journal.pone.0001764 (PMC2258150; doi:10.1371/journal.pone.0001764)
Supplement: Text S4 — Additional information for Figures 1– 3 (0.04 MB DOC) [file pone.0001764.s004.doc]

**Text S4. Additional information for Figures 1-3**

In few instances, the classification trees are expanded by incorporating branching nodes inferred from the additional coding-region sequences of [2,3,22,24], for which in most cases control-region information is unfortunately unavailable. In naming the sub-branches, we followed the scheme of [8] and use the principle that a valid code introduced in a publication first is preferred over a second name given later (in ignorance of the former). An exception would be made only in the case that a name given later has become most widely applied in the meantime, so that a shift back to the first name would rather create more confusion (as is e.g. the case with the generally employed name H2a of the subhaplogroup of H2 harboring the rCRS). For example, we attach the name C1a to the single known Asian branch of haplogroup C1 (Figure 1) following [22]. Many other haplogroup names for Asian and Native American haplogroups given in [22], however, violated previously published nomenclature without warning the reader of the change. For example, haplogroups B4, B4d, C4, D1, D4, D5, M8, and M8a (see [6] for earlier references) have been *de novo* baptized in [22], but the new codes have apparently not been followed elsewhere.
