# Supplementary material for: The Phylogeny of the Four Pan-American MtDNA Haplogroups: Implications for Evolutionary and Disease Studies
Source: PLoS One. 2008 Mar 12;3(3):e1764. doi: 10.1371/journal.pone.0001764 (PMC2258150; doi:10.1371/journal.pone.0001764)
Supplement: Text S5 — Additional references (0.04 MB DOC) [file pone.0001764.s005.doc]

**Text S5. Additional references**
