# Supplementary material for: The Phylogeny of the Four Pan-American MtDNA Haplogroups: Implications for Evolutionary and Disease Studies
Source: PLoS One. 2008 Mar 12;3(3):e1764. doi: 10.1371/journal.pone.0001764 (PMC2258150; doi:10.1371/journal.pone.0001764)
Supplement: Table S1 — Source of the complete mtDNA sequences (0.39 MB DOC) [file pone.0001764.s006.doc]

**Table S1. Source of the complete mtDNA sequences**

| **Sample ID**^a^ | **Haplogroup** | **Original ID** | **GenBank ID** | **Reference** |
| --- | --- | --- | --- | --- |
| 1 | A2a | Apache514 | EU095526 | [5] |
| 2 | X2a | Na3x | AY195787 | [31] |
| 3 | X2a | Oj2 | EU439939 | [1]; present study |
| 4 | C4c | Ijka72 | EU095543 | [5] |
| 5 | D2a | Aleut (#IX) | not available | [32] |
| 6 | D2a | Aleut (#I) | not available | [32] |
| 6 | D2a | Aleut (#II) | not available | [32] |
| 6 | D2a | Aleut (#III) | not available | [32] |
| 6 | D2a | Aleut (#IV) | not available | [32] |
| 6 | D2a | Aleut (#V) | not available | [32] |
| 6 | D2a | Aleut (#VI) | not available | [32] |
| 6 | D2a | Aleut (#VII) | not available | [32] |
| 7 | D4h3 | Cayapa600 | EU095531 | [5] |
| 8 | A2c | 332 | not available | [1] |
| 9 | A2c | 200 | not available | [1] |
| 9 | A2c | 241 | not available | [1] |
| 10 | A2c | 411 | not available | [1] |
| 11 | A2d1 | 418 | not available | [1] |
| 12 | A2d1 | A2-6-05 | DQ282433 | [33] |
| 13 | A2d1 | A2-6-03 | DQ282431 | [33] |
| 14 | A2d1 | A2-6-01 | DQ282429 | [33] |
| 15 | A2d1 | A2-6-02 | DQ282430 | [33] |
| 16 | A2d1 | A2-6-04 | DQ282432 | [33] |
| 17 | A2d2 | A2-1-02 | DQ282388 | [33] |
| 18 | A2d2 | 244 | not available | [1] |
| 18 | A2d2 | A2-3-14 | DQ282422 | [33] |
| 19 | A2d2 | A2-3-13 | DQ282421 | [33] |
| 20 | A2d | A2-3-10 | DQ282418 | [33] |
| 21 | A2d | 331 | not available | [1] |
| 22 | A2d | 376 | not available | [1] |
| 23 | A2 | Tor22 | EF079873 | Dominican Rep.; present study |
| 24 | A2e | 139 | not available | [1] |
| 25 | A2e | 400 | not available | [1] |
| 26 | A2e | 374 | not available | [1] |
| 27 | A2f | 12 | not available | [1] |
| 28 | A2f | 532 | not available | [1] |
| 29 | A2g | A2-1-06 | DQ282392 | [33] |
| 30 | A2g | 112 | not available | [1] |
| 30 | A2g | A2-1-05 | DQ282391 | [33] |
| 31 | A2g | 329 | not available | [1] |
| 32 | A2g | A2-2-05 | DQ282406 | [33] |
| 33 | A2h | 247 | not available | [1] |
| 34 | A2h | 171 | not available | [1] |
| 35 | A2i | 170 | not available | [1] |
| 36 | A2i | IAB_D6 | EU431080 | USA; present study |
| 37 | A2i | Patient | not available | [34] |
| 38 | A2j1 | A2-1-12 | DQ282398 | [33] |
| 39 | A2j1 | A2-1-07 | DQ282393 | [33] |
| 40 | A2j1 | 459 | not available | [1] |
| 41 | A2j1 | A2-2-03 | DQ282404 | [33] |
| 42 | A2j | A2-1-01 | DQ282387 | [33] |
| 43 | A2k1 | A2-1-14 | DQ282400 | [33] |
| 44 | A2k1 | A2-1-04 | DQ282390 | [33] |
| 45 | A2k1 | Wayuu24 | EU095552 | [5] |
| 46 | A2k | A2-3-09 | DQ282417 | [33] |
| 47 | A2 | 439 | not available | [1] |
| 48 | A2 | A2-1-24 | DQ282401 | [33] |
| 49 | A2 | A2-1-10 | DQ282396 | [33] |
| 49 | A2 | A2-1-13 | DQ282399 | [33] |
| 49 | A2 | A2-1-09 | DQ282395 | [33] |
| 49 | A2 | A2-1-11 | DQ282397 | [33] |
| 50 | A2 | #7 (Control) | not available | [12] |
| 51 | A2 | A2-5-01 | DQ282428 | [33] |
| 52 | A2 | A2-4-01 | DQ282424 | [33] |
| 53 | A2 | A2-4-03 | DQ282426 | [33] |
| 53 | A2 | A2-4-02 | DQ282425 | [33] |
| 53 | A2 | A2-4-04 | DQ282427 | [33] |
| 54 | A2 | IA_C3 | EU431081 | USA; present study |
| 55 | A2 | CanarAF_10 | AF382010 | [35] |
| 56 | A2 | 415 | not available | [1] |
| 57 | A2 | A2-3-03 | DQ282411 | [33] |
| 58 | A2 | A2-3-05 | DQ282413 | [33] |
| 59 | A2 | A2-3-04 | DQ282412 | [33] |
| 59 | A2 | A2-3-06 | DQ282414 | [33] |
| 59 | A2 | A2-3-08 | DQ282416 | [33] |
| 59 | A2 | A2-3-12 | DQ282420 | [33] |
| 59 | A2 | A2-3-01 | DQ282409 | [33] |
| 59 | A2 | A2-3-02 | DQ282410 | [33] |
| 59 | A2 | A2-3-07 | DQ282415 | [33] |
| 60 | A2 | A2-3-15 | DQ282423 | [33] |
| 61 | A2 | A2-3-11 | DQ282419 | [33] |
| 62 | A2 | IA_H4 | EU431082 | Canada; present study |
| 63 | A2 | Cayapa522 | EU095530 | [5] |
| 64 | A2 | 330 | not available | [1] |
| 65 | A2 | 120 | not available | [1] |
| 66 | A2 | 176 | not available | [1] |
| 67 | A2 | 184 | not available | [1] |
| 68 | A2 | A2-2-01 | DQ282402 | [33] |
| 69 | A2 | A2-2-02 | DQ282403 | [33] |
| 69 | A2 | A2-2-04 | DQ282405 | [33] |
| 69 | A2 | A2-2-06 | DQ282407 | [33] |
| 70 | A2 | A2-2-07 | DQ282408 | [33] |
| 71 | A2 | Arsario20 | EU095528 | [5] |
| 72 | A2 | A2-1-03 | DQ282389 | [33] |
| 73 | A2 | Kogui39 | EU095545 | [5] |
| 74 | A2 | Cayapa511 | EU095529 | [5] |
| 75 | A2 | Na5A | AY195786 | [31] |
| 76 | A2 | A2-1-08 | DQ282394 | [33] |
| 77 | A2 | Dogrib39 | EU095538 | [5] |
| 78 | B2a1a | B2-2-04 | DQ282444 | [33] |
| 79 | B2a1a | B2-2-06 | DQ282446 | [33] |
| 80 | B2a1 | B2-2-05 | DQ282445 | [33] |
| 81 | B2a1 | B2-2-03 | DQ282443 | [33] |
| 82 | B2a1 | B2-2-02 | DQ282442 | [33] |
| 83 | B2a1 | IA_E2 | EU431083 | USA; present study |
| 84 | B2a | B2-2-01 | DQ282441 | [33] |
| 85 | B2a | (4) Pi_26_27 | AF347001 | [36] |
| 86 | B2b | 419 | not available | [2,5] |
| 87 | B2b | Cayapa602 | EU095532 | [5] |
| 88 | B2c1 | B2-1-05 | DQ282438 | [33] |
| 89 | B2c1 | B2-1-06 | DQ282439 | [33] |
| 90 | B2c1 | B2-1-01 | DQ282434 | [33] |
| 91 | B2c1 | B2-1-07 | DQ282440 | [33] |
| 92 | B2c1 | B2-1-04 | DQ282437 | [33] |
| 93 | B2c | B2-1-03 | DQ282436 | [33] |
| 94 | B2c | Patient | not available | [37] |
| 95 | B2d | Na1B | AY195749 | [31] |
| 96 | B2d | Ngoebe14 | EU095546 | [5] |
| 97 | B2d | Wayuu7 | EU095550 | [5] |
| 98 | B2d | Wayuu17 | EU095551 | [5] |
| 99 | B2 | Coreguaje1-30 | EU095535 | [5] |
| 100 | B2 | IA_G1 | EU431084 | USA; present study |
| 101 | B2 | Waunana2-8 | EU095548 | [5] |
| 102 | B2 | Tor23 | EF079874 | Dominican Rep.; present study |
| 103 | B2 | B2-1-02 | DQ282435 | [33] |
| 104 | B2 | Native - Sinixt | EF648602 | Direct Submission |
| 105 | C1b1 | Na4C | AY195759 | [31] |
| 106 | C1b2a | C1-1-03 | DQ282449 | [33] |
| 107 | C1b2a | C1-1-02 | DQ282448 | [33] |
| 107 | C1b2a | C1-1-04 | DQ282450 | [33] |
| 107 | C1b2a | C1-1-05 | DQ282451 | [33] |
| 107 | C1b2a | C1-1-06 | DQ282452 | [33] |
| 107 | C1b2a | C1-1-08 | DQ282454 | [33] |
| 107 | C1b2a | C1-1-09 | DQ282455 | [33] |
| 107 | C1b2a | C1-1-10 | DQ282456 | [33] |
| 107 | C1b2a | C1-1-11 | DQ282457 | [33] |
| 107 | C1b2a | C1-1-12 | DQ282458b | [33] |
| 107 | C1b2a | C1-1-01 | DQ282447 | [33] |
| 108 | C1b2a | C1-1-07 | DQ282453 | [33] |
| 109 | C1b2 | CanarAF_09 | AF382009 | [35] |
| 110 | C1b3 | C1-2-06 | DQ282464 | [33] |
| 111 | C1b4 | C1-4-02 | DQ282475 | [33] |
| 112 | C1b4 | C1-4-03 | DQ282476 | [33] |
| 113 | C1b4 | IA_F1 | EU431085 | USA; present study |
| 114 | C1b5 | C1-2-03 | DQ282461 | [33] |
| 115 | C1b5 | C1-2-12 | DQ282469 | [33] |
| 116 | C1b | Wayuu4 | EU095549 | [5] |
| 117 | C1c2 | C1-2-11 | DQ282468 | [33] |
| 117 | C1c2 | C1-2-14 | DQ282471 | [33] |
| 117 | C1c2 | C1-2-08 | DQ282466 | [33] |
| 117 | C1c2 | C1-2-10 | DQ282467 | [33] |
| 118 | C1c2 | C1-2-04 | DQ282462 | [33] |
| 119 | C1c2 | C1-2-13 | DQ282470 | [33] |
| 120 | C1c | Arsario5 | EU095527 | [5] |
| 121 | C1c | Kogui12 | EU095544 | [5] |
| 122 | C1c | C1-2-01 | DQ282459 | [33] |
| 123 | C1c | C1-2-02 | DQ282460 | [33] |
| 124 | C1c | Tor24 | EF079875 | Dominican Rep.; present study |
| 125 | C1c | IA_A3 | EU431086 | Canada; present study |
| 126 | C1c | IA_A7 | EU431087 | USA; present study |
| 127 | C1c | C1-2-05 | DQ282463 | [33] |
| 128 | C1c | C1-2-07 | DQ282465 | [33] |
| 129 | C1d | C1-3-02 | DQ282473 | [33] |
| 129 | C1d | C1-3-03 | DQ282474 | [33] |
| 130 | C1d | C1-3-01 | DQ282472 | [33] |
| 131 | C1d | Coreguaje1-54 | EU095537 | [5] |
| 132 | C1d | (27) Wa_RML | AF347013 | [36] |
| 133 | C1d | (28) Wa_SPACH | AF347012 | [36] |
| 134 | D1a | (30) G_GRC150 | AF346984 | [36] |
| 135 | D1 | IA_G4 | EU431088 | Canada; present study |
| 136 | D1b | Tor25 | EF079876 | Dominican Rep.; present study |
| 136 | D1b | D1-1-03 | DQ282479 | [33] |
| 137 | D1b | D1-1-08 | DQ282484 | [33] |
| 138 | D1c | D1-1-02 | DQ282478 | [33] |
| 139 | D1c | D1-1-01 | DQ282477 | [33] |
| 139 | D1c | D1-1-05 | DQ282481 | [33] |
| 140 | D1d | D1-1-04 | DQ282480 | [33] |
| 141 | D1d | D1-1-09 | DQ282485 | [33] |
| 142 | D1 | Coreguaje1-31 | EU095536 | [5] |
| 143 | D1 | IA_F2 | EU431089 | USA; present study |
| 144 | D1 | D1-1-10 | DQ282486 | [33] |
| 145 | D1 | D1-1-06 | DQ282482 | [33] |
| 146 | D1 | D1-1-07 | DQ282483 | [33] |
| 147 | D1 | Na2D | AY195748 | [31] |
| 148 | D1 | D1-1-11 | DQ282487 | [33] |

^a^ These ID numbers correspond to those in Figures 1-3.

^b^ Coding-region information from [2,3]. Note that the indicated website (at the former company MitoKor), for downloading the complete mtDNA sequences, does no longer exist—and the user does not get redirected to the site where the data are now retrievable. The relocated source is http://mito546.securesites.net/science/30asianmtdnas.php. After downloading these sequences one is confronted with another obstacle: all 30 sequences have been misedited in the C stretches around position 310 in that one or two nucleotides C have been misplaced. Variant 317+C should be turned into 315+C instead, variant 315+CC into 309+C plus 315+C, and 315+CCC should become 309+CC plus 315+C. It is unclear whether these data represent the corrected coding-region sequences [3] since the general file at http://mito546.securesites.net/science/560mtdnasrevision.php still presents the flawed data [2], which thus also went into the mtDB database [4].
